# Reply to Guski, Schreckenberg, Schuemer, Brink and Stansfeld: Comment on Gjestland, T. A Systematic Review of the Basis for WHO’s New Recommendation for Limiting Aircraft Noise Annoyance. *Int. J. Env. Res. Pub. Health* 2018, *15*, 2717

**DOI:** 10.3390/ijerph16071105

**Published:** 2019-03-28

**Authors:** Truls Gjestland

**Affiliations:** SINTEF Digital, 0010 Oslo, Norway; truls.gjestland@sintef.no

**Keywords:** aircraft noise, annoyance, WHO recommendations

## Abstract

The European Regional Office of the World Health Organization (WHO, 2018) recently dramatically lowered its former (WHO, 2000) recommendations for cumulative aircraft noise exposure levels associated with risks of adverse public health effects. WHO’s recommendations, although lacking the force of law, are nonetheless of interest to aviation regulatory bodies and to the public at large. It is therefore important that WHO’s recent recommendations receive and withstand careful scrutiny. WHO’s (2018) recommendations are based on controversial assumptions, analyses and interpretations prepared by Guski et al. (2017). Gjestland (2018) identified a number of limitations of the opinions expressed by Guski et al. (2017). Guski et al. (2019) subsequently challenged some of Gjestland’s (2018) observations. This paper responds to the defenses offered by Guski et al. (2019) of the opinions expressed in their prior (2017) publication.

## 1. Introduction

The European Regional Office of the World Health Organization [1] recently dramatically lowered its former [2] recommendations for cumulative aircraft noise exposure levels associated with risks of adverse public health effects. WHO’s recommendations, although lacking the force of law, are nonetheless of interest to aviation regulatory bodies and to the public at large. It is therefore important that WHO’s recent recommendations receive and withstand careful scrutiny.

WHO [1] strongly recommends decreasing permissible noise exposure levels produced by aircraft to *L*_den_ = 45 dB to prevent adverse public health consequences. WHO’s newly identified noise exposure levels are an order of magnitude lower than those identified by WHO in 2000. WHO’s 2000 recommendations were not source-specific recommendations, but suggested a limit of *L*_pA,16h_ = 55 dB to avoid health effects mediated by serious annoyance. The corresponding L_den_ value would have been higher for a full 24- (rather than 16-h) day period. A source-specific correction for aircraft, however, would have moved WHO’s recommendation in the opposite direction. WHO’s new [1] recommendation thus represents a shift of about 10 dB from its former recommendation. A 10 dB reduction in the duration-corrected loudness of cumulative noise exposure (to *L*_den_ = 45 dB from *L*_pA,16h_ = 55 dB) represents “half as much noise” as WHO’s prior recommendation. This is a dramatic shift in the recommended “safe” limit on aircraft noise exposure, for which strong and reliable evidence is essential.

Gjestland’s [3] critical review of the evidence presented by Guski et al. [4] concluded that some of the referenced studies analyzed had not been conducted according to standardized methods, and that the samples of study respondents analyzed by Guski et al. [4] may have not been representative of a general airport population. Gjestland [3] therefore concluded that WHO’s 2018 recommendations for aircraft noise exposure limits were not based on fully reliable information.

## 2. On the Selection of Studies

Guski et al. [5] state that they did not consider information produced by the study at Trondheim airport by Gelderblom et al. [6] because the authors of that study did not provide equations or parameter estimates of their exposure-response relationships. This statement is incorrect and suggests that Guski et al. [5] are unfamiliar with assessments of noise-induced annoyance conducted by means other than ad hoc regression. The findings of the Trondheim airport study were expressed as a CTL (”Community Tolerance Level”) value, per ISO 1996:2016. CTL analysis yields a single number quantity that defines a complete exposure response function from 0% HA to 100% HA as explained in the international standard ISO 1996 [7]—precisely what Guski et al. requested. CTL analysis accomplishes this in a more parsimonious and parametrically efficient manner than regression analysis. It does so by accounting for more variance than logistic regression [8] without making independent empirical estimates of both slope and intercept of an exposure-response function.

## 3. On Non-Acoustic Factors

Univariate regression (that is, prediction of the prevalence of high annoyance from exposure levels alone) accounts for only about one third of the variance of individual annoyance responses. The prevalence of high annoyance in communities is also influenced by additional acoustic and non-acoustic factors, however. Acoustic factors include maximum levels, number of flights, fleet composition, and their respective distributions over time. Each of these factors also include errors of measurement and/or prediction. Non-acoustic factors include personal noise sensitivity and attitudes toward the noise source. In the aviation industry all “non- L_dn_ factors” are commonly referred to as “non-acoustic” (see, for instance ICAO White Papers on aircraft noise, symposia of the ANNA Group (Aircraft Noise Non-Acoustic), inter alia.

The latter (non-acoustic) factors are essentially useless for regulatory purposes, however, since they are unknown in advance, and cannot be used for a priori predictions of annoyance prevalence rates. Further, aircraft fly over all members of a community, not just those who may be more or less individually sensitive to aircraft noise exposure. As long as the preferred measure of community response to aircraft noise is the prevalence of a consequential degree of annoyance, non-acoustic influences on annoyance are simply free variables that contribute to errors of prediction. CTL analysis estimates the net effects of non-acoustic influences on annoyance in the aggregate, and by treating them systematically as deviations from an assumed growth rate of annoyance.

Gjestland [3] comments on a statement in the WHO 2018 recommendations regarding the variance in the results from noise annoyance studies that “acoustic factors (or rather L_den_ -based factors) may explain up to 33% of the variance, while the other two-thirds are explained by non-acoustic factors.” Guski et al. [5] insist that this is wrong, and without providing any references, claim that “the other two thirds are variance induced by non-acoustic factors (another 33%) and (another 33%) remaining non-explained (error) variance.” This is an idiosyncratic interpretation that departs from commonly used terminology.

Gjestland [3] notes in his critical review that “A procedure based on combining all responses from different surveys in this manner represents [a] simple way of analyzing data from aircraft noise annoyance surveys. It ignores the fact that only about one third of the variance in the response data is explained by the cumulative noise exposure […] and it effectively prohibits any possibility of studying the influence of non-acoustic factors […].” Guski et al. [5] reject this as nonsensical, a value judgment that is inappropriate in a scientific paper. It may be possible to infer the influence of non-acoustic factors from some social surveys, but a combined exposure-response function based on the findings of multiple surveys contains no useful information about the influence of different non-acoustic factors.

## 4. On Age Effects on Annoyance

Gjestland [3] identified inclusion of the findings of the HYENA study in the analyses of Guski et al. [4] as a key issue, because the HYENA study included non-standardized annoyance questions and a restricted age range (45–70 years) of respondents. In their 2017 analyses, Guski et al. state that “we can assume a certain bias towards higher annoyance. However, we did not have data to test this assumption.” In their comments on Gjestland’s critique, however, this doubt has disappeared, and Guski et al. [5] dismiss any possibility that the limited age range may have had an influence on the annoyance response. This evolved position of Guski et al. [5] is not supported by existing evidence [9]. The HYENA researchers themselves state that “Our results may not be fully comparable to the EU curve, because the HYENA study annoyance was assessed in the limited age range of 45–70-year-old subjects” [10] (page 1175). The original researchers’ concerns are not shared by Guski et al. [5].

One of the inclusion criteria for the meta-analysis of Guski et al. [4] was “studies including members of the general population.” Including the findings of a study of reactions of a limited age group in their meta-analysis is a clear a violation of this criterion. Including studies with samples who are not representative of the general population in a meta-analysis casts doubt on the generalizability of the findings of the meta-analysis to public health analyses.

## 5. Additional Reasons for Excluding Results from the HYENA Study

The annoyance questions of most modern noise surveys comply with ICBEN [11] recommendations. This was also one of the criteria listed by Guski et al. [4] for selecting studies to be included in their meta-analysis. The HYENA study used separate annoyance questions for daytime and nighttime annoyance. Guski et al. [4] analyzed only the daytime response to represent “annoyance in general”, as specified by ICBEN. Guski et al. [5] do not comment on this issue, but in an earlier comment Guski, Schuemer and Schreckenberg [12] admit this decision can be questioned. They cite, without testing, an assumption made by Babisch et al. [10] that “the overall annoyance is mostly determined by the annoyance during the daytime.” The inclusion by Guski et al. [4] of the results of the HYENA study, which contributed 28% of the WHO full dataset, thus appears to be based on an unsupported assumption.

To avoid known and unknown biases, the opinions of each and every member of a target population must have an equal probability of representation in social survey findings. This is commonly provided by random selection of a current and exhaustive list of the target population compiled into a sample frame. Examination of the sampling methods of the HYENA study at Heathrow reveal disturbing deviations from EPSEM (equal probability of selection of elements) sampling. Eligible residents were contacted by mail by researchers at Imperial College. They received an information pack that requested their participation. At the same time a letter from HACAN, a noise interest group at Heathrow, was sent to its members and other voluntary organizations, urging them to participate in the survey. A follow-up letter is not uncommon in a mail survey but urging members of a select group (in this case, people opposed to the airport), invites bias. HACAN’s letter further solicits self-selection for participation in the study as follows: “If you have not received an information pack….and would be interested in taking part, please contact (xx at Imperial College).” (See Supplementary material). Encouraging self-selection into an opinion sample is inconsistent with universally recognized procedures for the conduct of surveys.

Likewise, there is reason to question the adequacy of the exposure estimates at several of the HYENA airports. Babisch et al. cite aircraft noise levels as low as *L*_Aeq 24h_ = 11 dB (Stockholm) and *L*_Aeq 24h_ = 22 dB (Milan). Such aircraft noise levels are only marginally credible as modeling estimates, much less as acoustic measurements. Guski et al. [4] convert these estimates to *L*_den_ by applying a correction factor of 2.6414 dB [13]. These values seem to indicate that the researchers rely heavily on existing models to describe the aircraft noise exposure, with little evident concern for the limitations of aircraft noise modeling software, and for the credibility of predicted exposure values. No existing aircraft noise prediction programs can yield credible exposure predictions at such low levels (indicating a very long distance from the source). Applying a correction accurate to “one ten-thousandth of a decibel”, to a number that has been predicted by a model that yields output values with a standard error of at least 1–2 dB [14], makes no sense at all.

## 6. On the Use of the Community Tolerance Level (CTL) Approach

CTL analysis assumes that the rate of growth of annoyance with transportation noise exposure is fully controlled by the duration-adjusted loudness of the exposure. This growth function is anchored to the noise-axis (*x*-axis) by a community-specific annoyance decision criterion, expressed directly in units of decibels [8]. CTL analysis assumes a fixed function with a plausible and logical explanation for its annoyance-inducing response, rather than estimates of the slope and intercept (two separate parameters) of arbitrary fitting functions derived by regression techniques. Regression curves lack any physical or psychological explanation or interpretation.

The meta-analysis of Guski et al. [3] completely ignores the use of CTL analysis. Pointing to the wide range of individual exposure response curves in their report, they express doubts that a curve with a single slope can fit the findings of all of the surveys they selected for their meta-analysis. (They do not seem to appreciate that translating an *e*^(−A/m)^ prediction laterally along the abscissa displays different segments of the ogival growth function that do not appear parallel when pinned at different exposure values to the abscissa.) Their disbelief is further conditioned on their view that univariate logistic regression is some form of gold standard for the derivation of exposure-effect relationships.

**Figure 1:**
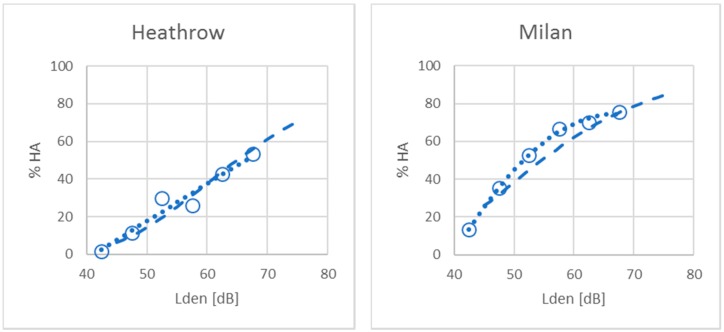

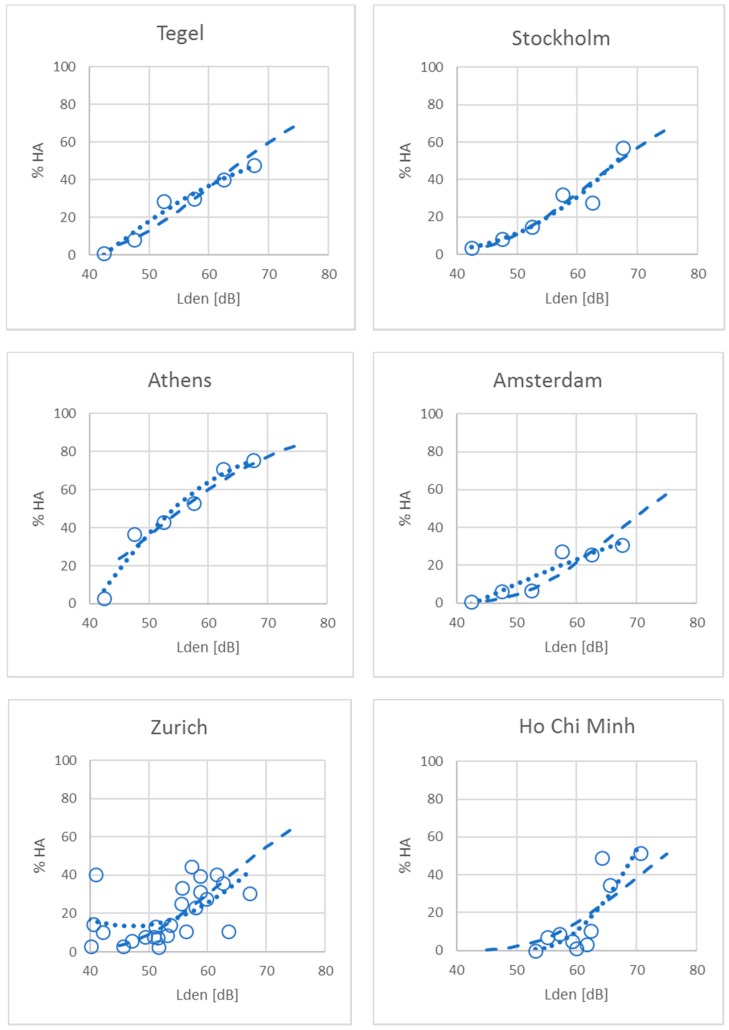

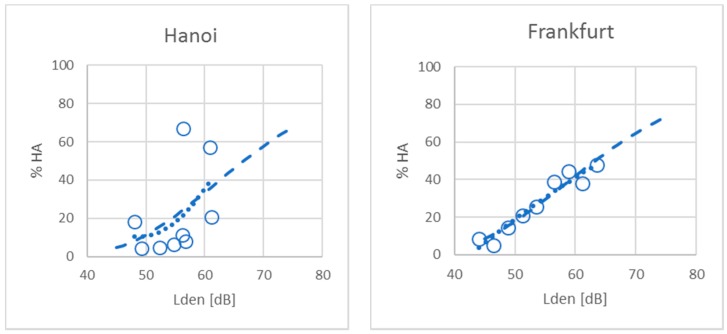
Survey data from individual airports with corresponding CTL curve (dashed) and 2nd order polynomial regression function (dotted).

A closer look at the actual response data reveals a different picture. Response data expressed as pairs of % HA and noise level have been found for all the surveys in the WHO full dataset except for the 2002 Amsterdam survey by Breugelmans et al. The results from these surveys are plotted in the panels of Figure 1, together with the CTL function and individually calculated 2nd order polynomial functions. The goodness-of-fit to the observed datapoints can be expressed by the coefficient of determination, r^2^. Table 1 shows the value r^2^ for the CTL function and the polynomial regression functions preferred by Guski et al.

Table 1 shows no meaningful differences in the coefficients of determination between two-parameter (slope and intercept) fits to the survey data produced by univariate multiple regression, and a CTL fit of the data to a single parameter, fixed growth function for annoyance prevalence rates. Further empirical evidence of the central assumption of CTL analysis has been published by Fidell et al. [8] and Schomer et al. [15] among others. Contrary to the views of Guski et al. [5], the form of the exposure-response relationship is well described by a more parsimonious fixed function than that produced by logistic regression.

## 7. Conclusions

The comments by Guski et al. [5] do not infirm the main conclusions of Gjestland’s [3] critical review of the bases of WHOs new recommendations for limiting aircraft noise. Guski et al. [5] offer no further justification for reliance on non-standardized annoyance questions, limited age-range for the respondents, and potential self-selection biases in the HYENA study. Even if all the surveys analyzed by Guski et al. [4] had been conducted by irreproachable methods, the fact that a similar analysis of a different (and larger) set of survey data [3] yields a very different result clearly indicates that the findings of Guski et al. [4] are not representative of community response to aircraft noise around airports in general.

As has been shown in Gjestland’s original paper [3], and further documented in the current rebuttal to the comments of Guski et al. [5], WHO’s new recommendations for limiting aircraft noise are based on questionable evidence.

## Figures and Tables

**Table 1:** Coefficient of determination for CTL curves and statistical regression functions.

Airport	CTL r^2^	Regress r^2^
Amsterdam, 2003	0.81	0.88
Athens, 2003	0.94	0.96
Berlin, Tegel, 2003	0.92	0.97
Heathrow, 2003	0.92	094
Milan, 2003	0.95	0.99
Stockholm, 2003	0.90	0.91
Zurich, 2001	0.23	0.26
Ho Chi Minh, 2008	0.72	0.73
Hanoi, 2009	0.22	0.24
Da Nang, 2011	0.23	0.25
Frankfurt, 2005	0.93	0.93

## Supplementary Materials

The following are available online at https://www.mdpi.com/1660-4601/16/7/1105/s1, Copy of letter from HACAN.

## References

[1] World Health Organization (2018). Environmental Noise Guidelines for the European Region.

[2] World Health Organization (2000). Guidelines for Community Noise.

[3] Gjestland T. (2018). A systematic review of the basis for WHO’s new recommendation for limiting aircraft noise annoyance. Int. J. Environ. Res. Public Health.

[4] Guski R., Schreckenberg D., Schuemer R. (2017). WHO Environmental Noise Guidelines for the European Region. A systematic review on environmental noise and annoyance. Int. J. Environ. Res. Public Health.

[5] Guski R., Schreckenberg D., Schuemer R., Brink M., Stansfeld S. (2019). Comment on Gjestland, T. A systematic review of the basis for WHO’s new recommendation for limiting aircraft noise annoyance. Int. J. Environ. Res. Public Health.

[6] Gelderblom F., Gjestland T., Granøien I.T. The impact of civil versus military aircraft noise on noise annoyance. Proceedings of the INTERNOISE-2014.

[7] ISO (2016). ISO 1996-1. Acoustics—Description, Measurement and Assesment of Environmental Noise—Part 1: Basic Quantities and Assessment Procedures.

[8] Fidell S., Mestre V., Schomer P., Berry B., Gjestland T., Vallet M., Reid T. (2011). A first principles model for estimating the prevalence of annoyance with aircraft noise exposure. J. Acoust. Soc. Am..

[9] Van Gerven P., Vos H., Van Boxtel M., Janssen S., Miedema H. (2009). Annoyance from environmental noise across the lifespan. J. Acoust. Soc. Am..

[10] Babisch W., Houthuijs D., Cadum E., Katsouyanni K., Velonakis M., Dudley M., Maron H.-D., Swart W., Breugelmans O., Bluhm G. (2009). Annoyance due to aircraft noise has increased over the years—Results of the HYENA study. Environ. Int..

[11] Fields J., de Jong R., Gjestland T., Flindell I., Job R., Kurra S., Lercher P., Vallet M., Yano T., Guski R. (2001). Standardized general-purpose. J. Sound Vib..

[12] Guski R., Schuemer R., Schreckenberg D. (2018). Comments on preprint. Int. J. Environ. Res. Public Health.

[13] Brink M. (2015). Conversion of Transportation Noise Exposure Metrics, Working Paper.

[14] Fidell S., Schomer P. (2007). Uncertainties in measuring aircraft noise and predicting community response to it. Noise Control Eng. J..

[15] Schomer P., Mestre V., Fidell S., Berry B., Gjestland T., Vallet M., Reid T. (2012). Role of community tolerance level in predicting the prevalence of the annoyance of road and rail noise. J. Acoust. Soc. Am..

