# Supplementary material for: Reply to Guski, Schreckenberg, Schuemer, Brink and Stansfeld: Comment on Gjestland, T. A Systematic Review of the Basis for WHO’s New Recommendation for Limiting Aircraft Noise Annoyance. Int. J. Env. Res. Pub. Health 2018, 15, 2717
Source: Int J Environ Res Public Health. 2019 Mar 28;16(7):1105. doi: 10.3390/ijerph16071105 (PMC6480183; doi:10.3390/ijerph16071105)
Supplement: Supplementary file 1 [file ijerph-16-01105-s001.pdf]

Telephone & fax: 020 8876 0455
Website: www.hacan.org.uk
Email: info@hacan.org.uk

OVERFLOWN & OVERL[illegible]

June 2005

TO VOLUNTARY ORGANISATIONS

AIRPORTS AND HEALTH STUDY, Imperial College, London

We are supporting this important research project taking place in 4 outer London Boroughs and are inviting you and your members to take part.

Dr Lars Jarup, of Imperial College is the Lead Investigator in a 6 nation study includ[illegible] The Netherlands, Greece, Italy, Germany and Sweden. We welcome this initiative w[illegible] is the first time such a large study has taken place looking at ways that airport noise, [illegible] noise and pollution might affect our health. The study has been approved by the Wel[illegible] Medical Research Ethics Committee and is funded by the European Union.

If you or any of your friends and neighbours would like to take part in this very interesting and valuable study, please turn to the details given overleaf. More inform[illegible] can be viewed at www.hyena.eu.com

With many thanks,

John Stewart

John Stewart
Chairman

AIRPORTS AND HEALTH STUDY

A team from Imperial College London is looking at ways that airport noise, road noise, and pollution might affect health. They are looking for 1,000 men and women aged between 45 and 70 who have lived in the area for at least five years.

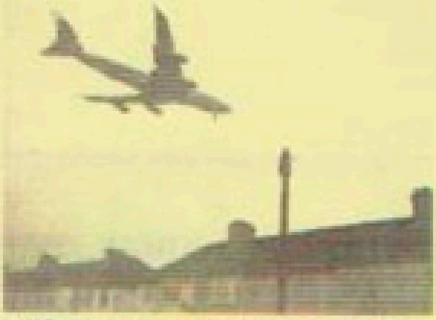

Taking part in the study involves a qualified nurse making a single visit to you at home on a day and time that suits you. During the visit the nurse will ask you some set questions about your health, your lifestyle and your feelings about noise and pollution. **You do not have to be bothered by noise to take part in this study. We would like to hear from you whatever your views.**

Residents of EALING, HARROW, HOUNSLOW & HILLINGDON are invited to take part in this study.

Participants are selected by postcode and you may receive or have received an invitation. **If you have an invitation pack from Imperial College London they would very much like to hear from you.**

If you have **not** received an information pack but meet the study criteria and would be interested in taking part, please telephone **Marie-Louise Dudley** on **Freephone 0800 089 5523** for further information

Marie-Louise Dudley, Scientific Coordinator Airports and Health Study
Department of Epidemiology and Public Health, Imperial College London.
Upper Ground Floor, Praed Street Wing, St Mary's Campus, Norfolk Place, London. W2 1PG
Tel: 020 7594 3322 Fax: 020 75943193 FREEPHONE: 0800 089 5523
Email: m.dudley@imperial.ac.uk Website: www.imperial.ac.uk
